# Bridging the Resolution Gap in Recurrent Prostate Cancer Imaging: Quantum Dots as PSMA-Targeted Optical Probes

**DOI:** 10.1021/acsomega.6c01161

**Published:** 2026-09-02

**Authors:** Yugmee Gidiya, Jiya Chaudhary, Annafew Biswas, Mandy Liao, Marine Ramaroson, Ken-ichiro Kamei

**Affiliations:** † Nanobiotechnology Class, Program of Bioengineering, Division of Engineering, New York University Abu Dhabi, Abu Dhabi 129188, United Arab Emirates; ‡ Program of Biology, Division of Science, New York University Abu Dhabi, Abu Dhabi 129188, United Arab Emirates; § Department of Biomedical Engineering, Tandon School of Engineering, New York University, Brooklyn, New York 11201, United States; ∥ Department of Biology, Faculty of Arts & Science, New York University, New York, New York 10003, United States; ⊥ Institute for Integrated Cell-Material Sciences, Kyoto University Institute for Advanced Study, Kyoto 606-8501, Japan; # Department of Pharmaceutics, Wuya College of Innovation, Shenyang Pharmaceutical University, Shenyang 110016, China; ¶ Joint International Research Laboratory of Intelligent Drug Delivery Systems, Ministry of Education, Shenyang 110016, China

## Abstract

Recurrent prostate cancer (RPC) remains a major clinical challenge driven by residual and disseminated tumor cells exhibiting pronounced molecular and spatial heterogeneity. Prostate-specific membrane antigen (PSMA)-targeted positron emission tomography enables sensitive whole-body detection of recurrent lesions; however, its millimeter-scale spatial resolution and limited multiplexing capacity constrain the characterization of cellular heterogeneity and micrometastatic disease. Quantum dots (QDs) are nanoscale optical probes with high brightness, exceptional photostability, and narrow, size-tunable emission spectra, enabling multiplexed molecular imaging at subcellular resolution. When functionalized with PSMA-targeting ligands, QDs facilitate the sensitive and spatially resolved visualization of PSMA expression, supporting the detection of heterogeneous and low-abundance tumor cell populations. Among QD platforms, cadmium-based systems offer superior optical performance for multiplexed imaging, whereas cadmium-free and carbon-based QDs provide improved biocompatibility and greater translational potential. Nevertheless, challenges related to toxicity, biodistribution, and clearance remain significant barriers to clinical application. This Review highlights QDs as complementary optical probes that may bridge the resolution gap between whole-body imaging and cellular-scale analysis, with particular relevance for preclinical research, ex vivo tissue interrogation, and intraoperative imaging. Collectively, QD-based strategies provide a conceptual framework for enhancing molecularly specific, high-resolution visualization in RPC imaging workflows.

## Graphical abstract

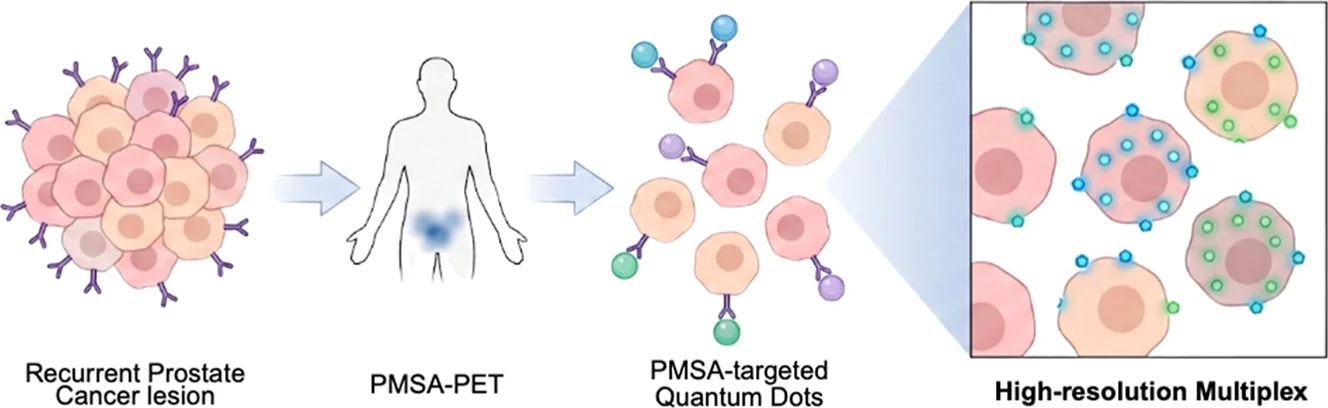

## 1 Introduction

Prostate cancer (PC) relapse following definitive therapy remains a major barrier to durable disease control and patient survival. Despite advances in surgery, radiotherapy, and systemic treatments, a substantial proportion of patients develop biochemical recurrence, often driven by residual or disseminated tumor cells that evade initial therapy.^1–3^ Early detection of recurrent prostate cancer (RPC) is therefore critical, as timely intervention can significantly influence therapeutic outcomes and disease progression.

Molecular imaging has transformed the clinical management of RPC, particularly with the advent of prostate-specific membrane antigen (PSMA)-targeted positron emission tomography (PET)^4,5^ (Figures 1 and 2). PSMA PET imaging enables highly sensitive whole-body detection of recurrent lesions, even at low prostate-specific antigen (PSA) levels, thereby supporting contemporary restaging strategies. However, this intrinsic millimeter-scale spatial resolution limits the ability to resolve cellular heterogeneity and micrometastatic disease.^4,6^ Consequently, small clusters of recurrent cells and phenotypically diverse subpopulations may remain undetected or insufficiently characterized.

**Figure 1:**
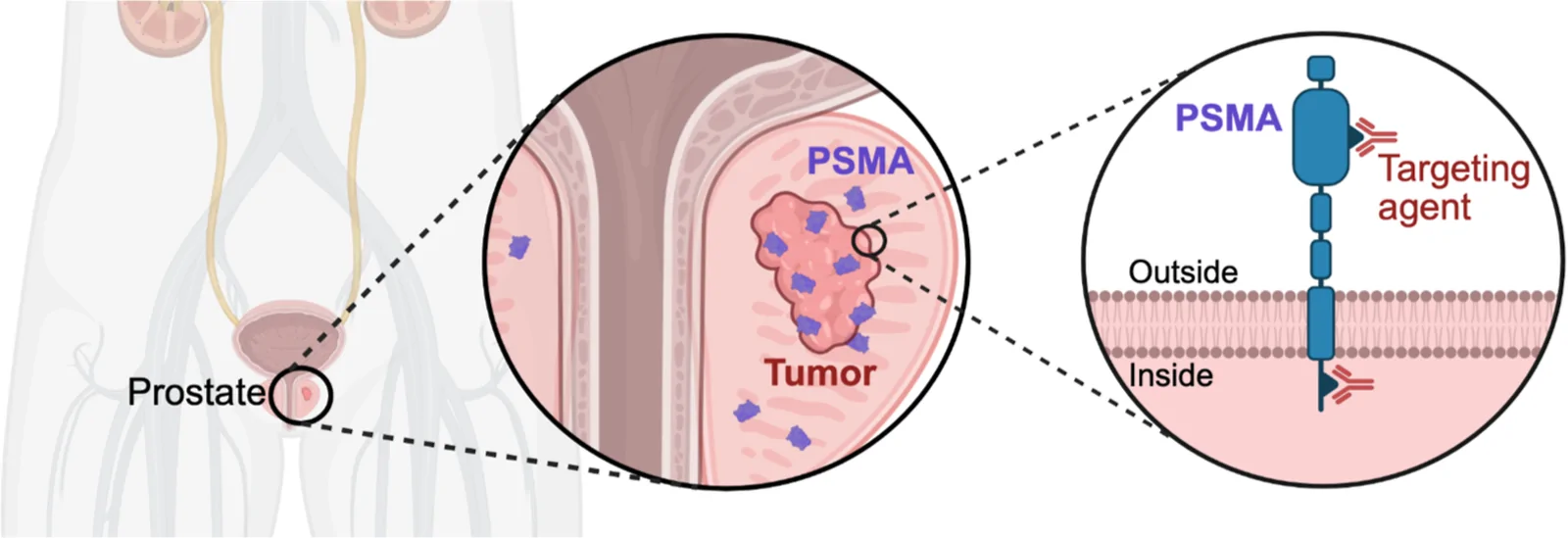
Prostate-specific membrane antigen (PSMA) as a molecular target in prostate cancer (PC). Schematic illustration of PSMA expression and targeting in PCs. Left panel: anatomical location of the prostate and a representative tumor lesion enriched in PSMA-positive cells. The central magnified view highlights the PSMA localization on the tumor cell membrane. The right panel depicts the membrane topology of PSMA, emphasizing its extracellular domain, which is accessible to targeting agents including antibodies, peptides, and small-molecule ligands. This accessibility enables the selective binding of PSMA-targeted imaging and therapeutic probes, forming the basis for PSMA-directed diagnostic imaging and targeted cancer therapy workflows. Created with BioRender (Kamei, K., 2026; https://BioRender.com/gw3my7l).

**Figure 2:**
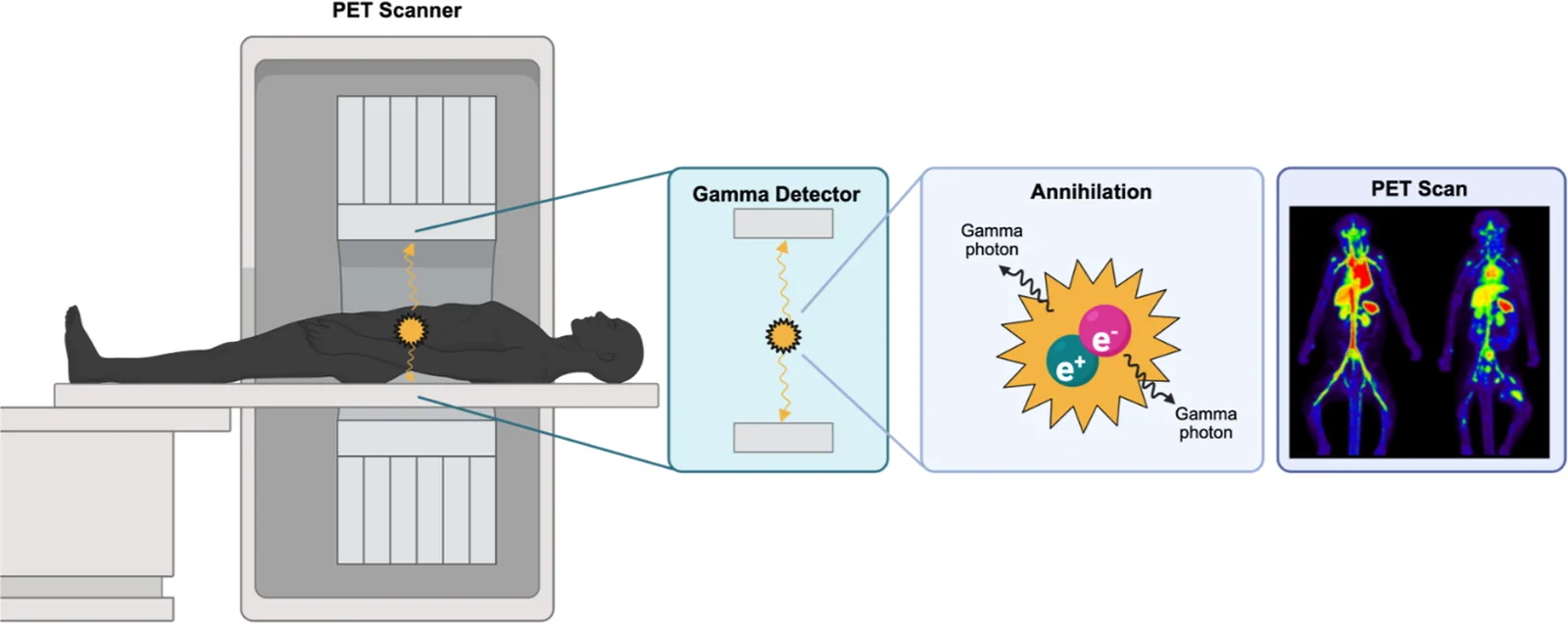
Principle of positron emission tomography (PET) imaging. Schematic representation of the fundamental operating principle of PET. Following systemic administration, a positron-emitting radiotracer accumulates in target tissues based on its biological specificity. Emitted positrons (e^+^) rapidly interact with nearby electrons (e^–^), resulting in annihilation events that produce two gamma photons emitted in nearly opposite directions (∼180° apart). These coincident photons are detected by opposing detector elements within the PET scanner. By registering multiple coincidence events, the system reconstructs a three-dimensional image reflecting the spatial distribution of the radiotracer. This process enables sensitive whole-body visualization of molecular and metabolic activity, forming the basis of PET imaging in cancer detection and therapeutic guidance. Created with BioRender (Kamei, K., 2026; https://BioRender.com/5fxnv4a).

These limitations underscore a broader challenge in cancer imaging: a mismatch between whole-body diagnostic sensitivity and the need for high-resolution, molecularly specific visualization at tissue and cellular scales.^7^ Although PET, magnetic resonance imaging (MRI), and ultrasound are effective for macroscopic lesion detection and anatomical assessment, they are not designed to capture the biological complexity underlying recurrence, including spatial heterogeneity and adaptive resistance.

Optical imaging offers a complementary approach for biomarker-driven visualization of molecular targets.^8–13^ In addition to high spatial resolution, it enables multiplexed detection, defined as the simultaneous measurement of multiple molecular targets within the same sample using spectrally distinct probes. However, its clinical and translational impact in RPC has been constrained by the limitations of conventional fluorescent probes, including photobleaching (irreversible signal loss under prolonged illumination), broad emission spectra, and suboptimal signal-to-noise performance in complex biological environments.

Quantum dots (QDs) are semiconductor nanocrystals whose optical properties are governed by quantum confinement, enabling size-dependent tuning of emission wavelengths. QDs have emerged as nanoscale optical probes capable of overcoming several limitations of conventional fluorophores.^14–18^ Their high brightness, exceptional photostability, and narrow emission spectra enhance sensitivity and spectral separation (Figure 3). Moreover, their surfaces can be functionalized with antibodies, peptides, or small-molecule ligands to enable selective targeting of cancer-associated biomarkers such as PSMA (Figure 4). While cadmium-based QDs have historically demonstrated superior optical performance, recent advances have expanded the development of cadmium-free alternatives with improved biocompatibility and translational profiles.^19,20^

**Figure 3:**
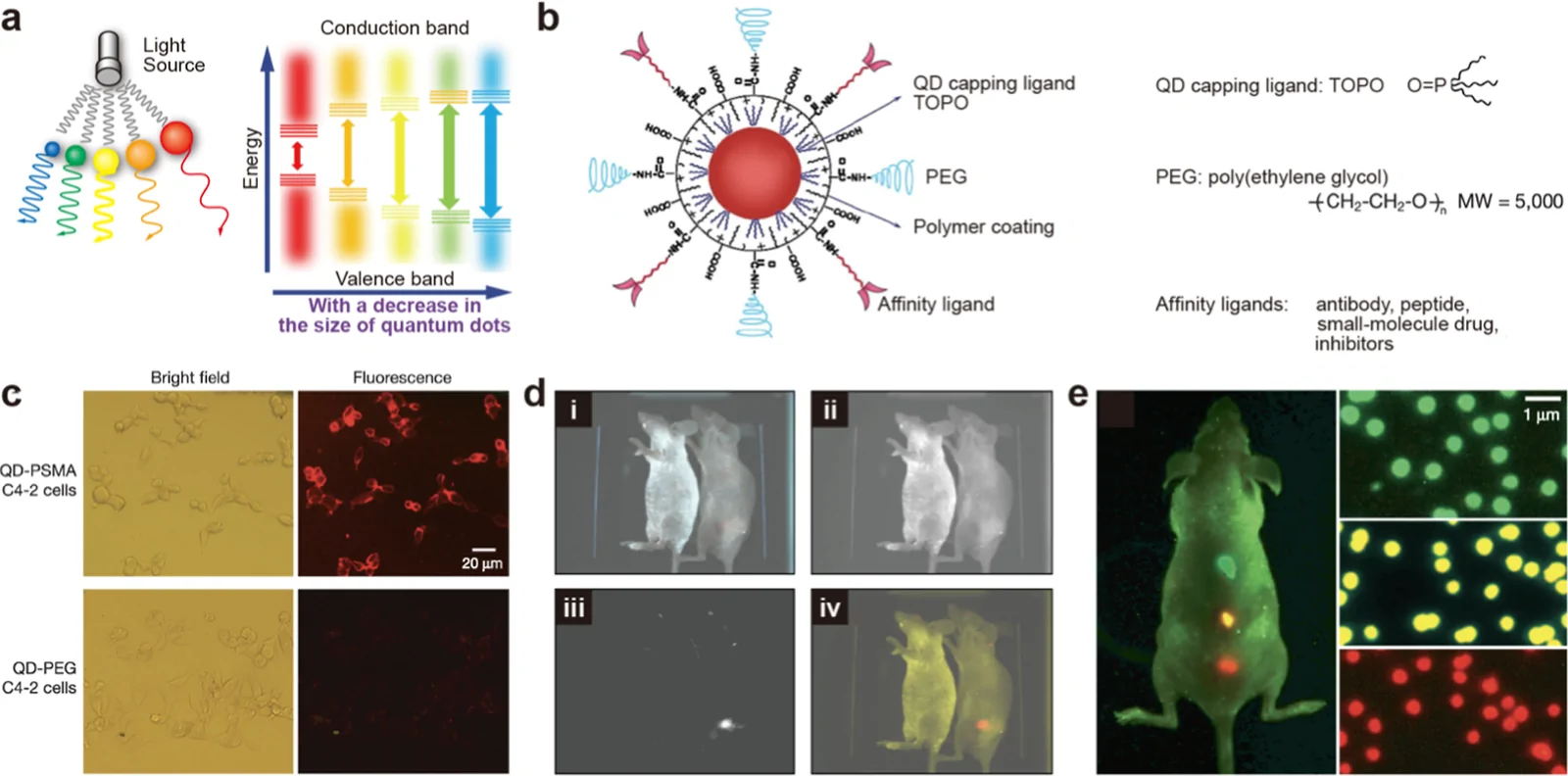
Optical properties, surface functionalization, and imaging capabilities of QDs. (a) Schematic illustration of size-dependent emission of QDs arising from quantum confinement, in which decreasing particle size results in a blue shift of emission wavelength. (b) Representative surface architecture of functionalized QDs, including core nanocrystal, capping ligands, polymer coatings (e.g., polyethylene glycol), and affinity ligands (e.g., antibodies, peptides, or small molecules) that enable selective targeting of biological molecules. (c) Bright-field and fluorescence micrographs of C4–2 cells incubated with QD-PSMA conjugates or PEG-coated QDs (QD–PEG). Strong fluorescence is observed in cells treated with QD–PSMA, whereas minimal signal is detected in QD–PEG controls, indicating specific targeting. (d) In vivo spectral imaging of QD–PSMA conjugates in mice bearing C4–2 tumor xenografts. Localized fluorescence signals correspond to tumor accumulation, while control animals show no detectable signal. Spectral unmixing is shown as (i) original image; (ii) autofluorescence; (iii) QD signal; and (iv) merged image. (e) Multicolor imaging capability of QDs demonstrated by simultaneous detection of spectrally distinct QD-encoded microbeads in vivo. Together, these panels illustrate the tunable emission, targeting versatility, and multiplexing capability of QDs, supporting their application as high-resolution optical probes. Panel (a) adapted from Debnath et al.^67^ Panels (c–e) reproduced from Gao et al.,.^14^ Copyright 2004 Springer Nature.

**Figure 4:**
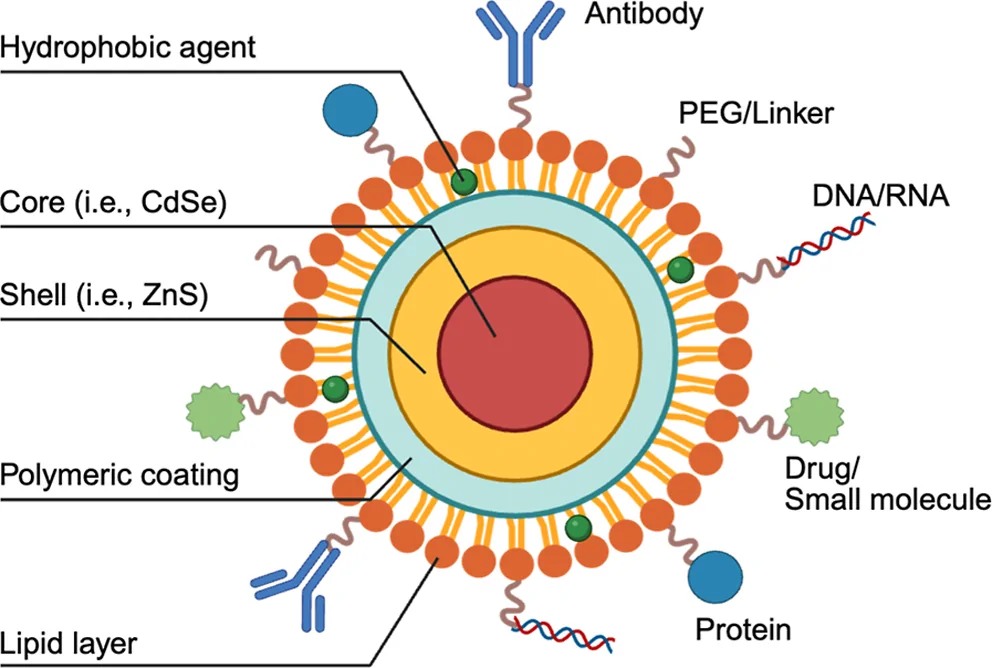
Surface functionalization strategy of QDs for targeted imaging and multifunctional applications. Schematic representation of a core–shell QD with modular surface functionalization. The semiconductor core (red) is encapsulated by an intermediate inorganic shell (yellow; e.g., ZnS), which enhances photostability and quantum yield while reducing core-associated toxicity. An outer hydrophilic coating (light blue), such as silica or polymer/poly­(ethylene glycol), provides colloidal stability and biocompatibility in biological environments. The QD surface is further functionalized via linker chemistries with diverse moieties, including targeting ligands or antibodies (blue) for biomarker recognition (e.g., PSMA), nucleic acids (red/blue helix) for sensing or tracking, and small molecules or drugs (green) for diagnostic or therapeutic integration. This hierarchical architecture enables QDs to function as versatile nanoplatforms for targeted imaging, multiplexed detection, and integrated diagnostic–therapeutic (theranostic) applications. Created with BioRender (Kamei, K., 2026; https://BioRender.com/gzu8w0c).

In this Review, we position QDs as complementary tools within PSMA-directed RPC imaging workflows rather than replacements for established clinical modalities. We integrate perspectives from cancer biology, imaging science, and nanomaterials engineering to critically evaluate how QDs may address current limitations in spatial resolution, biomarker multiplexing, and mechanistic interpretation. Particular emphasis is placed on material performance, targeting strategies, toxicity, and biodistribution considerations for clinical translation.

## 2 RPC Poses a Multiscale Imaging Challenge

RPC represents a biologically and clinically distinct disease state from that of primary PC. Following definitive treatment, residual or disseminated tumor cells may persist in a dormant or slow-proliferative state for prolonged periods before manifesting as local recurrence or distant metastasis, most commonly in bone and lymphatic tissues. These recurrent cell populations frequently exhibit altered molecular phenotypes, increased adaptability, and pronounced heterogeneity, reflecting the selective pressures imposed by prior therapy and the tumor microenvironment.

Clinically, recurrence is often first indicated by rising serum PSA levels.^21,22^ Although PSA is a sensitive marker of biochemical relapse, it lacks spatial resolution and provides no information regarding lesion location, tumor burden, or biological heterogeneity. This limitation creates a diagnostic gap between the systemic biochemical evidence of recurrence and the precise anatomical or molecular localization of disease.

At the molecular level, RPC cells commonly exhibit an elevated expression of PSMA, a transmembrane glycoprotein involved in folate metabolism and cellular signaling (Figure 1). PSMA overexpression has been widely exploited for targeted imaging and therapy, establishing it as a central biomarker in recurrent and advanced diseases. However, PSMA expression is heterogeneous across tumor cells and metastatic sites. Micrometastatic lesions, small clusters of recurrent cells, and phenotypically distinct subpopulations may exhibit variable or low PSMA expression, thereby complicating the detection and characterization.

Collectively, these features highlight a fundamental multiscale challenge in RPC imaging. Disease progression is driven by processes operating at the cellular and microlesion levels, whereas clinical detection and monitoring rely predominantly on imaging modalities optimized for organ-level or whole-body visualization. Bridging this scale mismatch is essential for improving early detection, resolving intratumoral heterogeneity, and enabling more precise biomarker-guided intervention strategies.

## 3 Limitations of Current Imaging Modalities and the Rationale for Optical Probes

Current imaging modalities have substantially improved RPC clinical detection and management of RPC; however, each approach exhibits intrinsic limitations that become particularly evident at early or micrometastatic stages (Figure 5). PSMA-targeted PET combined with computed tomography (PSMA PET/CT) enables sensitive whole-body detection but is fundamentally constrained by spatial resolution on the order of several millimeters, limiting its ability to resolve small clusters of cancer cells or intralesional molecular heterogeneity. MRI provides excellent soft-tissue contrast and high anatomical detail^1,23^ but relies primarily on structural differences rather than molecular specificity, thereby reducing sensitivity for dispersed or biologically inactive lesions. Ultrasound imaging offers real-time visualization and broad accessibility^24^ but remains largely restricted to structural assessment and lacks molecular sensitivity.

**Figure 5:**
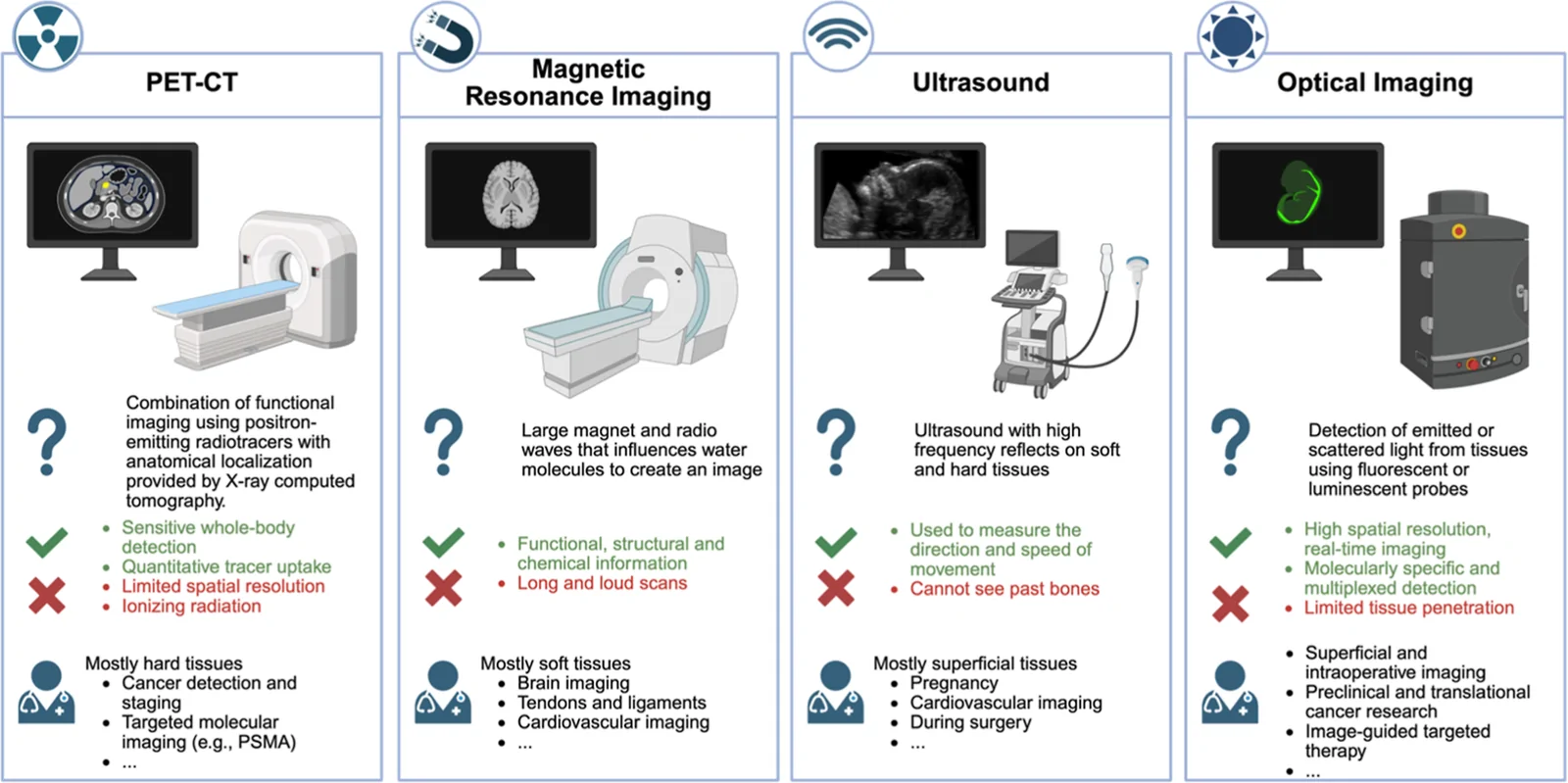
Comparison of major imaging modalities used in cancer detection and therapy guidance. Schematic overview of PET–computed tomography (PET–CT), MRI, ultrasound, and optical imaging, highlighting their respective imaging principles, strengths, limitations, and typical applications. PET–CT integrates molecularly targeted radiotracer imaging with anatomical localization, enabling sensitive whole-body detection but with millimeter-scale spatial resolution. MRI provides high soft-tissue contrast and functional information without ionizing radiation, although acquisition times are relatively long. Ultrasound enables real-time, accessible imaging for superficial tissues and procedural guidance but is limited by acoustic penetration and lacks molecular specificity. Optical imaging allows high-resolution, real-time, and molecularly specific visualization using fluorescent or luminescent probes; however, its application is constrained by limited tissue penetration. Collectively, these modalities illustrate complementary capabilities across spatial scales, supporting integrated imaging workflows for cancer detection and therapy guidance. Created with BioRender (Kamei, K., 2026; https://BioRender.com/ygaz3ji).

Taken together, these limitations reveal a consistent theme: current modalities are optimized for macroscopic detection and anatomical characterization but are not designed to interrogate molecular features or cellular-scale heterogeneity. This constraint is particularly significant in RPC, where disease progression and treatment resistance are driven by spatially and phenotypically diverse cellular populations.

Optical imaging approaches provide an orthogonal strategy based on targeted probe–biomarker interactions.^25,26^ Rather than replacing established modalities, optical imaging is most effective in settings requiring localized molecular readouts, including ex vivo tissue analysis, intraoperative visualization, and mechanistic studies in model systems.

However, the performance of optical imaging is strongly dependent on the probe characteristics. Conventional organic fluorophores are limited by broad emission spectra, photobleaching during prolonged imaging, and relatively low brightness, which collectively constrain the sensitivity and spectral discrimination. These limitations have historically restricted the translational impact of fluorescence-based approaches on cancer imaging.

QDs (typically 2–10 nm in a diameter) address these limitations through their distinctive photophysical properties (Table 1).^14–18^ Their narrow emission spectra enable improved spectral separation, while high brightness supports robust signal detection in complex biological environments (Figure 3a). In addition, surface functionalization with targeting ligands^27–30^ enables selective interrogation of biomarkers such as PSMA and facilitates analysis of tumor heterogeneity and biomarker coexpression (Figure 3b).^18,31,32^

**Table 1:** Comparative Summary of QD (QD) Types for Biomedical Applications

quantum dot type	composition	key advantages for biomedical use	primary drawbacks/concerns	example cancer application
cadmium-based QDs	CdSe or CdTe cores (often with ZnS shell)^19,20,68^	high brightness and size-tunable emission for multicolor imaging	potential toxicity from Cd^2+^ ion release and ROS generation	molecular tumor imaging (e.g., antibody-conjugated QDs); intraoperative lymph node mapping
		well-established surface chemistry for ligand conjugation	long-term accumulation in organs (e.g., liver and spleen)	
indium phosphide QDs	InP core (typically ZnS shell)^64^	cadmium-free alternative with emission in the visible–NIR range [69]	lower initial quantum yield than cadmium-based QDs (improving with engineering)	targeted tumor imaging (e.g., antibody-conjugated InP/ZnS QDs); combined imaging and miRNA delivery
		improved stability via core–shell engineering	long-term biocompatibility requires further evaluation	
		potential for theranostic applications when functionalized		
CuInS_2_QDs (ternary QDs)	CuInS_2_ core (often alloyed or coated with ZnS or Ag)^43–45^	cadmium- and lead-free composition	moderate photoluminescence quantum yield compared with Cd-based QDs	in vivo tumor imaging; bimodal MRI–optical probes (e.g., gadolinium-chelated systems)
		NIR emission suitable for deeper tissue imaging	dose-dependent oxidative stress reported in some studies	
		potential for multimodal imaging (e.g., MRI–optical)		
carbon quantum dots (CQDs)	carbon-based nanodots (amorphous or graphitic core)^29,46,47^	low toxicity and absence of heavy metals	limited intrinsic NIR emission without modification	cell imaging and drug delivery; experimental photothermal applications when doped
		good aqueous dispersibility and facile surface functionalization	lower brightness per particle than semiconductor QDs	
		tunable photoluminescence and photostability		
graphene quantum dots (GQDs)	nanoscale graphene fragments^27,50,51^	high biocompatibility and photostability	lower quantum yield relative to semiconductor QDs	theranostic platforms combining imaging and PTT/PDT; targeted drug delivery
		NIR absorption/emission potential	variability in size and surface properties depending on synthesis	
		intrinsic photothermal and photodynamic capabilities		
silicon quantum dots	Si nanocrystals (∼2–5 nm), often oxide- or polymer-coated^54,55^	metal-free and biodegradable into nontoxic byproducts	lower photoluminescence efficiency relative to conventional QDs	targeted imaging (e.g., ligand-functionalized Si QDs); potential intraoperative imaging agents
		favorable biocompatibility	susceptibility to oxidation affecting emission stability	
		tunable photoluminescence with surface passivation		
perovskite QDs	lead-halide (e.g., CsPbBr_3_) or lead-free (e.g., Cs_2_AgBiBr_6_) perovskites^15,57,58^	high brightness and narrow emission peaks	lead-related toxicity concerns in common formulations	experimental tumor imaging; photodynamic therapy; surface-engineered systems for targeted delivery
		tunable optical properties via composition and size	instability under moisture and light exposure	
		emerging multimodal imaging and therapeutic potential	limited *in vivo* validation to date	
		emerging use in multimodal imaging and therapy (fluorescence imaging, PTT, and PDT)		

In the context of RPC, these combined attributes position QDs as scale-bridging probes that link whole-body detection to cellular-level analysis.^33^ This capability is particularly relevant for integrating diagnostic imaging with mechanistic investigations of disease progression.^29,30^

Clinically established PSMA-targeted PET tracers, including 68Ga-68 PSMA-11 ^34,35^ and ^18^F-DCFPyL,^35,36^ represent the current standard for detecting recurrent disease due to their high sensitivity, deep tissue penetration, and validated clinical performance.^35^ While these agents enable whole-body imaging, they remain inherently limited by millimeter-scale spatial resolution and an inability to resolve intralesional heterogeneity or perform multiplexed molecular analysis.^4,6^ In contrast, QD-based optical probes operate at the tissue and cellular scale,^37^ enabling high-resolution visualization and multiplexed detection of multiple biomarkers within the same sample. However, limited imaging depth and their current preclinical status constrain their use in systemic in vivo applications.^38^ Accordingly, QDs should be considered complementary to PSMA PET imaging rather than replacements, addressing distinct gaps in spatial resolution and molecular complexity (Table 2).

**Table 2:** Comparative Analysis of PSMA-Targeted PET Imaging and QD (QD)-Based Optical Probes across Spatial, Functional, and Translational Dimensions

feature	PSMA PET imaging	QD-based optical imaging
spatial resolution	millimeter scale	micrometer to cellular scale
imaging depth	whole-body	limited (millimeter–centimeter range)
multiplexing capability	not available	enabled (simultaneous detection of multiple targets)
clinical status	clinically established	preclinical/early translational stage

## 4 PSMA-Targeted QD Strategies for RPC

The optical and functional advantages of QDs translate directly into targeted imaging strategies when these nanomaterials are conjugated to PSMA-directed ligands. PSMA represents a particularly attractive molecular target (Figure 1, Table 3).^1,4,6,39^ It is a transmembrane glycoprotein that is overexpressed in recurrent, advanced, and metastatic PC while exhibiting limited expression in most normal tissues. This differential expression profile has established PSMA as a central biomarker for molecular imaging and has driven the development of PSMA-directed probes across multiple imaging modalities.

**Table 3:** Summary of Experimental Demonstrations of PSMA-Targeted QD (QD) Probes^a^

QD platform	targeting moiety	model	key finding	reference
CdSe/ZnS QD–A10 aptamer		LNCaP (PSMA^+^) vs PC3 (PSMA^–^) cells (in vitro)	selective labeling of PSMA^+^ cells; doxorubicin loading enabled combined imaging and drug delivery via bi-FRET	^40^
CdSe/ZnS QD–anti-PSMA Ab	J591 monoclonal antibody	LNCaP xenograft mice; in vitro binding assays	preferential accumulation in PSMA^+^ tumors; ∼4-fold higher fluorescence compared with nontargeted controls	^39^
CdSe/ZnS QD–PSMA inhibitor	urea-based small-molecule inhibitor	LNCaP and DU145 cells; confocal imaging	PSMA-dependent internalization demonstrated by colocalization; subcellular trafficking visualized	^42^
InP/ZnS NIR QD	anti-PSMA targeting moiety	prostate tumor xenograft mice	sustained tumor fluorescence up to 24 h postinjection; lymph node drainage visualized	^43^
CuInS_2_/ZnS QD (cadmium-free)	urea-based PSMA-targeting inhibitor	LNCaP cells (confocal); PSMA^+^ spheroids	selective PSMA-mediated uptake confirmed; cadmium-free formulation with NIR-compatible emission	^42^

a Abbreviations: Ab, antibody; NIR, near-infrared; PSMA, prostate-specific membrane antigen; and QD, quantum dot.

QDs can be readily functionalized with PSMA-targeting moieties, including antibodies, antibody fragments, peptides, aptamers, and small-molecule ligands.^44^ Such conjugation enables selective binding to PSMA-expressing PC cells while leveraging the high brightness and photostability of QDs for sensitive optical detection. QDs typically exhibit extinction coefficients in the range of 10^5^–10^6^ M^–1^ cm^–1^—1 to 2 orders of magnitude higher than those of conventional organic fluorophores—and can achieve quantum yields of 50–80% under optimized conditions, contributing to markedly enhanced signal intensity.^45^ In the context of RPC, where tumor cells may be sparse, dispersed, or present as micrometastatic foci, these properties are particularly advantageous for detecting low-abundance targets.^43,46^

Emerging experimental studies provide proof-of-concept evidence that QD-based probes can enhance molecular imaging performance beyond conventional approaches. In preclinical models, QDs conjugated with tumor-targeting ligands have demonstrated high-contrast visualization of cancer cells both in vitro and in vivo, including the detection of small cell clusters that are difficult to resolve with standard imaging modalities.^14,47^ PSMA-targeted QDs, in particular, exhibit selective labeling of PC cells, with specificity validated by differential uptake in PSMA-expressing versus PSMA-negative cell lines in both in vitro and in vivo systems (Figure 3c,d, respectively).^14,40,42^ Compared with conventional fluorophores, QDs provide greater signal stability and brightness, enabling prolonged imaging durations and more reliable detection of low-abundance targets.^47–49^

A representative example is illustrated in Figure 3 (adapted from Gao et al.).^14^ In vitro, QD–antibody conjugates demonstrate highly selective binding to PSMA-expressing C4–2 cells, with minimal nonspecific interaction in PSMA-negative controls, confirming strong molecular specificity. In vivo, systemic administration in tumor-bearing mouse models results in preferential accumulation of QDs within PSMA-positive xenografts, with clear fluorescence delineation of tumor margins relative to surrounding tissue. The high signal-to-background contrast reflects the combined effects of targeted molecular recognition and the intrinsic photophysical properties of QDs, including high quantum yield and resistance to photobleaching. Sustained fluorescence intensity over extended imaging periods further supports their suitability for longitudinal imaging applications.

Collectively, these findings indicate not only qualitative tumor localization but also quantitative improvements in imaging contrast and specificity relative to surrounding tissue. A key advantage of QD-based PSMA imaging lies in its ability to resolve molecular heterogeneity at spatial scales that are inaccessible to conventional clinical imaging. RPC is frequently characterized by heterogeneous PSMA expression both within individual lesions and across metastatic sites. High-resolution optical imaging with QDs enables direct visualization of this heterogeneity, supporting detailed analysis of target distribution, cellular binding patterns, and subpopulation structure—information that is difficult to extract from whole-body imaging modalities that rely on signal averaging over millimeter-scale volumes.

Beyond single-target detection, QDs also enable multiplexed molecular analysis relevant to recurrent and treatment-resistant PC (Figure 3e).^8–14^ In this context, PSMA can be evaluated alongside additional biomarkers associated with disease progression, therapeutic resistance, or tumor–microenvironment interactions. This multiplexing capability is particularly valuable for dissecting complex phenotypes and understanding how heterogeneous cell populations contribute to recurrence.

From a translational perspective, PSMA-targeted QDs are best viewed as complementary optical tools rather than replacements for established clinical imaging modalities. Their most immediate applications include high-resolution preclinical studies, *ex vivo* analysis of patient-derived tissues, intraoperative or margin-assessment imaging, and integration with advanced *in vitro* systems such as organoids^50^ and organ-on-chip platforms.^51–53^ In these settings, molecularly specific and spatially resolved information can support target validation, therapeutic development, and mechanistic insight with limited systemic exposure.

Despite these promising attributes, current evidence remains largely preclinical, and direct clinical comparisons with established modalities such as PSMA PET/CT are limited. Furthermore, key translational challenges—including biodistribution, clearance, and potential off-target accumulation—must be addressed before clinical implementation.

Overall, PSMA-targeted QD strategies illustrate how nanomaterial-based optical probes can extend PC imaging beyond whole-body detection toward high-resolution, localized analysis of PSMA biology (Table 3). In this capacity, QDs provide a complementary approach that bridges the gap between macroscopic imaging and cellular level characterization of RPC.

## 5 Surface Chemistry of QDs: Ligand Engineering, Functionalization Strategies, and the Path to PSMA Targeting

The QD surface is not merely a structural boundary but the primary interface governing interactions with biological systems. Control over surface chemistry is therefore inseparable from the design of functional PSMA-targeted imaging probes (Table 4). Upon exposure to physiological environments, uncoated or poorly passivated QDs rapidly adsorb serum proteins, forming a dynamic “protein corona” that alters colloidal stability, shifts cellular uptake pathways from receptor-mediated endocytosis to nonspecific macropinocytosis, redirects biodistribution toward mononuclear phagocyte system (MPS) organs, and sterically hinders targeting ligands from engaging PSMA on tumor cells.^54,55^ To address these effects, three principal engineering strategies have been developed: modification of native ligands in situ, exchange with water-soluble ligands, and encapsulation of the QD core within a biocompatible shell.

**Table 4:** Representative Pharmacokinetic Parameters of QD Platforms in Preclinical Rodent Models^a^

QD type	surface coating	HD size (nm)	blood *t*1/2 (h)	primary accumulation	reference
CdSe/ZnS (semiconductor)	none (TOPO-capped)	∼15–20	<0.25	liver, spleen	^49^
CdSe/ZnS	PEG-750	∼20–25	∼0.7	liver, spleen	^49^
CdSe/ZnS	PEG-5000	∼25–35	∼3	liver, spleen (reduced uptake)	^49^
Ag_2_Se (NIR semiconductor)	PEG	∼14	∼0.4	liver, lung; tumor (EPR effect)	^56^
ultrasmall CdSe	zwitterionic cysteine	<5.5	∼0.5	predominantly renal clearance (urinary excretion)	^57^
CQDs/GQDs (carbon-based)	native –COOH/–OH groups	∼3–8	∼0.5–1.5	liver, lung; partial renal clearance	^58^

a Abbreviations: HD, hydrodynamic diameter; *t*1/2, blood elimination half-life; TOPO, trioctylphosphine oxide; PEG, polyethylene glycol; EPR, enhanced permeability and retention; CQD, carbon quantum dot; GQD, graphene quantum dot.

The choice among these strategies depends on the native surface state, which is dictated by the synthesis route. Organic-phase high-temperature synthesis—commonly used for indium phosphide (InP)/ZnS, CdSe/ZnS, and CuInS_2_/ZnS QDs with high photoluminescence quantum yields—produces surfaces capped with hydrophobic ligands such as trioctylphosphine oxide (TOPO), trioctylphosphine (TOP), or oleylamine.^59^ These ligands preserve optical quality by minimizing surface trap states and ensuring colloidal stability in nonpolar solvents but render the particles incompatible with aqueous biological environments. In contrast, aqueous-phase synthesis yields QDs functionalized with short thiol ligands (e.g., mercaptopropionic acid [MPA], glutathione, or cysteine) or bearing carboxyl and amine groups. These particles are inherently water-dispersible but typically exhibit lower photoluminescence quantum yields due to less effective surface passivation and increased susceptibility to oxidative ligand displacement.

To render organically synthesized QDs biocompatible, ligand exchange or encapsulation is required. In ligand exchange, native hydrophobic ligands are replaced with bifunctional, water-soluble molecules such as dihydrolipoic acid–polyethylene glycol (DHLA–PEG) conjugates,^60^ MPA,^61^ or zwitterionic ligands.^62,63^ These ligands anchor to the QD surface via thiol, phosphonate, or imidazole groups while presenting a hydrophilic exterior. Zwitterionic coatings are particularly advantageous for translational applications, as their near-neutral surface charge suppresses protein adsorption, reduces corona formation, and minimizes accumulation in liver and spleen.^49^ Notably, when the hydrodynamic diameter is maintained below approximately 5.5 nm,^64^ zwitterionically coated QDs undergo renal clearance via glomerular filtration, an important consideration for reducing long-term organ retention.^65–67^ Encapsulation provides an alternative approach in which the native ligand–core interface is preserved. In this strategy, TOPO-capped QDs are surrounded by amphiphilic polymers or phospholipid–PEG micelles, maintaining optical properties while introducing a hydrophilic outer layer for biological compatibility. Although this approach offers excellent stability and broad material compatibility, it typically increases hydrodynamic size (>15–20 nm), which may limit renal clearance and alter biodistribution.

Among the introduced functional groups, PEG plays a central dual role.^60^ PEG coatings provide a “stealth” layer that reduces opsonization and MPS uptake while simultaneously serving as a scaffold for conjugation of targeting ligands via terminal functional groups. Bifunctional PEG derivatives bearing carboxyl (PEG-COOH), primary amine (PEG-NH_2_), or maleimide groups enable conjugation of aptamers, antibodies, or small-molecule inhibitors through established bioconjugation chemistries.^41,68,69^ However, PEG design involves trade-offs: increasing chain length or grafting density enhances protein resistance but also increases hydrodynamic size and may reduce accessibility of conjugated ligands, thereby lowering effective targeting density.^70–72^ In near-infrared (NIR)-II QD systems, surface modification has been shown to independently influence quantum yield, colloidal stability, and targetability, underscoring the importance of surface chemistry optimization.^73^

The choice of bioconjugation chemistry for PSMA targeting depends on the nature of the targeting ligand. Three principal strategies have been demonstrated. The A10 RNA aptamer, which binds the extracellular domain of PSMA, can be conjugated via streptavidin–biotin interactions or direct amine coupling, and its functionality can be extended by doxorubicin intercalation to generate theranostic QD–aptamer constructs.^40^ Anti-PSMA monoclonal antibodies such as J591 are typically conjugated through EDC/NHS-mediated coupling or maleimide–thiol chemistry, with the latter enabling site-directed attachment that preserves antigen-binding fragment (Fab) orientation.^39^ Small-molecule urea-based PSMA inhibitors derived from PSMA-617 scaffolds provide compact targeting ligands with functional groups for direct covalent attachment, enabling minimal hydrodynamic size. The biodistribution of such constructs has been characterized using X-ray fluorescence imaging and inductively coupled plasma mass spectrometry.^42^ A comprehensive overview of QD bioconjugation strategies is provided by Sobhanan et al.^59^

Surface chemistry considerations are highly material-specific. In metallic QDs, ZnS shell passivation reduces leakage of cytotoxic ions (e.g., Cd^2+^ or In^3+^) and suppresses surface trap states that quench photoluminescence.^59^ InP QDs present an additional challenge due to formation of an indium oxide layer (In_2_O_3_) upon air exposure, which must be removed prior to efficient thiol ligand binding.^74^ CuInS_2_ QDs exhibit intrinsic copper vacancies that act as nonradiative trap states, necessitating optimized shell engineering. In contrast, carbon-based QDs and graphene QDs inherently possess surface functional groups (e.g., carboxyl, hydroxyl, amine), enabling direct bioconjugation without prior modification.^25,58,75^ Silicon QDs, initially hydrogen-terminated, require surface stabilization via hydrosilylation to generate alkyl- or silanol-terminated surfaces suitable for biological applications.^76–78^

Collectively, these considerations highlight that surface chemistry is a critical determinant of QD performance, influencing optical properties, targeting efficiency, biodistribution, and the translational potential. Careful selection and optimization of surface functionalization strategies are therefore essential for the successful development of PSMA-targeted QD probes for high-resolution molecular imaging.

## 6 QD Material Platforms: Optical Performance versus Translational Feasibility

Selection of an appropriate QD platform for PC imaging requires balancing optical performance with translational feasibility (Tables 1 and 4). While high brightness, narrow emission bandwidth, and photostability are essential for sensitive and multiplexed imaging, material composition, surface chemistry, and in vivo behavior ultimately determine biocompatibility, biodistribution, clearance, and regulatory viability. Consequently, no single QD platform is optimal across all applications. Instead, each material class presents a distinct combination of advantages and constraints. A comparative evaluation of these trade-offs is therefore more informative than identifying a universally “best” system and enables rational alignment of QD materials with specific imaging contexts within targeted cancer therapy workflows.

### 6.1 Cadmium-Based QDs as Optical Performance Benchmark

Cadmium selenide (CdSe)/ZnS core–shell nanocrystals represent the most extensively characterized semiconductor QD platform in biomedical imaging, and their photophysical properties define the benchmark against which alternative fluorescent nanomaterials are evaluated. The canonical structure comprises a CdSe semiconducting core (2–8 nm in diameter) overcoated with a thin ZnS shell (typically 1–3 monolayers) of a wider band gap, which passivates surface trap states that would otherwise quench emission. This core–shell architecture enables photoluminescence quantum yields of 60–90%, substantially exceeding those of conventional organic fluorophores (typically 10–40%). Photostability is likewise markedly superior: Ballou et al.^49^ demonstrated that PEGylated CdSe/ZnS QDs retain fluorescence for 4 months following intravenous administration in mice, a duration that organic fluorophores cannot approach under comparable conditions. Together with high molar extinction coefficients, these properties make CdSe/ZnS QDs particularly well suited for single-molecule tracking and long-term in vivo imaging applications.^79^

The emission wavelength of CdSe/ZnS QDs is precisely tunable through the control of core size. Because the semiconductor band gap scales inversely with the core diameter via quantum confinement, emission can be tuned approximately 480 nm (∼2 nm core) to 640 nm (∼6 nm core). Extension into the NIR region is achieved through compositional engineering, including type II heterostructures (e.g., CdSe/ZnSe, CdTe/CdSe) and alloyed cores that shift emission beyond 700 nm. Kim et al.^73^ demonstrated the practical utility of this approach using type II CdSe-based QDs emitting at approximately 900 nm for real-time imaging of sentinel lymph nodes at a depth of 1 cm in large animals during surgery, using an injected dose of 400 pmol and an excitation fluence rate of 5 mW cm^–2^. CdSe/ZnS QDs also exhibit narrow emission bandwidths (full-width at half-maximum, 25–35 nm), and particles of different sizes can be excited with a single excitation wavelength. This property enables simultaneous multiplexed imaging of multiple molecular targets with minimal spectral crosstalk. For example, Peng et al.^80^ used spectrally distinct QD probes to covisualize multiple stromal biomarkers in gastric cancer tissue microarrays, demonstrating multiplexed detection capabilities that are not achievable with conventional immunohistochemistry or single-color fluorophores. Such multiplexing is directly relevant to RPC, where colocalization of PSMA with androgen receptor splice variants or other resistance-associated markers could inform treatment stratification.

Despite these advantages, clinical translation of CdSe/ZnS QDs remains constrained by cadmium-related toxicity and long-term in vivo retention. A comprehensive analysis by Oh et al.^81^ encompassing 1741 cell viability data points from 307 studies identified surface properties (including shell composition and ligand identity), particle size, and exposure duration as the primary determinants of toxicity. Surface passivation strategies, particularly ZnS shell coating combined with PEG functionalization, can substantially mitigate the cytotoxic effects. For instance, Peng et al.^82^ reported that PEG-coated CdSe/ZnS QDs exhibited no detectable cytotoxicity in HepG2 liver cells at concentrations of up to 100 nM, whereas free Cd^2+^ ions were toxic at much lower thresholds. However, the long-term fate of cadmium released from degraded nanocrystals in vivo remains incompletely understood, and regulatory approval of cadmium-containing imaging agents for human use has not yet been achieved. Consequently, CdSe/ZnS QDs continue to serve as the optical benchmark for QD-based imaging, with cadmium-free alternatives evaluated on the basis of their ability to approach comparable quantum yield, spectral tunability, and photostability while offering improved safety profiles.

### 6.2 Indium Phosphide QDs

InP is a III–V semiconductor with a zinc-blende crystal structure and a bulk band gap of approximately 1.35 eV. In contrast to cadmium selenide, the In–P bond exhibits a predominantly covalent character, conferring greater chemical robustness and reduced susceptibility to oxidative degradation relative to II–VI counterparts.^74^ When coated with a ZnS shell (InP/ZnS), the surface trap states are effectively passivated, enabling photoluminescence quantum yields of 40–70%.^83^ However, emission bandwidths remain broader than those of CdSe/ZnS QDs, with full-width at half-maximum values of approximately 40–60 nm compared with 25–35 nm for cadmium-based systems. Brown et al.^84^ reported InPZn/ZnS QDs with quantum yields of ∼50% and tunable emission via variation of the zinc-to-indium ratio but observed that the ZnS shell prepared by conventional methods exhibited reduced stability relative to CdSe/ZnS systems, resulting in emission degradation under ambient conditions. This reduced shell stability provides a mechanistic basis for the lower photostability of InP QDs and represents a key limitation for multiplexed or longitudinal imaging applications.

InP QDs have been applied in targeted cancer imaging and theranostic platforms. Wu et al.^74^ functionalized NIR InP QDs with a vascular endothelial growth factor receptor 2 monoclonal antibody and a miR-92a inhibitor, enabling receptor-mediated uptake, tumor accumulation, and three-dimensional NIR imaging of leukemia xenografts in vivo. Similarly, Zeng et al.^85^ incorporated InP/ZnS QDs into graphene oxide-based nanocomposites loaded with miR-122, demonstrating combined NIR imaging and apoptosis induction in drug-resistant hepatoma models, both in vitro and in vivo. The use of NIR emission reduces photon scattering relative to that of visible wavelengths, facilitating improved tissue penetration. Collectively, these studies indicate that InP QDs can be effectively functionalized with antibodies and nucleic acid payloads for targeted imaging and therapeutic delivery in cancer model systems.

The in vivo safety and biodistribution of InP/ZnS QDs were evaluated in multiple rodent studies. Li et al.^86^ reported that intravenously administered InP/ZnS QDs bearing hydroxyl, amino, or carboxyl surface groups accumulated predominantly in the liver and spleen for up to 28 days. At low doses, no overt histopathological abnormalities were observed; however, higher doses of carboxyl-functionalized QDs induced acute inflammatory responses and alterations in hepatic and cardiac biomarkers. A complementary study demonstrated that hydroxyl- and amino-terminated InP/ZnS QDs increased kidney injury molecule-1 (KIM-1) expression and upregulated apoptosis-related genes (Bax, caspase-3, −7, and −9), indicating potential renal toxicity.^87^ These findings underscore the central role of surface chemistry in determining organ-specific toxicity profiles. In addition, Zhang et al.^88^ showed that cytotoxic responses to InP/ZnS and CdSe/ZnS QDs varied by cell type, with reduced viability observed in HeLa but not in ML-1 cells. This effect correlated with increased uptake and lysosomal trafficking, suggesting that intracellular transport dynamics rather than composition alone are critical determinants of toxicity.

For PSMA-targeted optical imaging in RPC, InP/ZnS QDs represent a cadmium-free alternative with a moderate quantum yield and established covalent conjugation chemistries. Their in vivo toxicity profile at low doses is generally more favorable than that of CdSe/ZnS QDs, although their long-term safety remains under investigation. However, broader emission bandwidths limit multiplexing capacity relative to cadmium-based systems, and the reduced photostability of the ZnS shell constrains applications requiring prolonged or repeated imaging. Quantitative evaluation of hepatic and renal retention following imaging-relevant dosing will be essential for advancing InP QDs toward clinical translation.

### 6.3 Copper Indium Sulfide QDs (CuInS_2_/ZnS QDs)

CuInS_2_ is a ternary I–III–VI_2_ chalcopyrite semiconductor with a tetragonal crystal lattice and a bulk band gap of approximately 1.5 eV. In contrast to binary II–VI (CdSe) or III–V (InP) QDs, its optical properties are dominated by defect-mediated processes rather than purely by quantum confinement. Photoluminescence arises primarily from donor–acceptor pair (DAP) recombination involving copper vacancy states within the band gap. This mechanism produces two defining spectroscopic features: a large Stokes shift (100–300 nm or 0.2–0.8 eV) and a broad emission bandwidth (full-width at half-maximum [fwhm], 80–120 nm). The large Stokes shift reduces reabsorption and inner-filter effects at higher probe concentrations, whereas the broad emission bandwidth limits spectral resolution and thus constrains multiplexed imaging. Emission can be tuned from approximately 550 nm to beyond 950 nm by controlling particle size (∼2–5 nm) and Cu/In stoichiometry, spanning the visible to NIR range relevant for tissue imaging. ZnS shell passivation increases the photoluminescence quantum yield from ∼5–20% in bare cores to 30–85% and improves both colloidal stability and photostability in biological environments.^89–91^

Early in vivo demonstrations highlight the translational potential of this platform. Pons et al.^92^ reported real-time fluorescence imaging of regional lymph nodes in mice using water-dispersible CuInS_2_/ZnS QDs emitting at ∼800 nm. Notably, lymph node inflammation occurred only at doses approximately 10-fold higher than those required to induce comparable effects with cadmium-containing QDs, suggesting an improved safety margin. Liu et al.^93^ further demonstrated targeted imaging by coating CuInS_2_/ZnS QDs with an amphiphilic bovine serum albumin–poly­(ε-caprolactone) conjugate and functionalizing with cyclic RGD peptide for selective visualization of integrin αvβ3-overexpressing tumor cells. The resulting nanoprobes exhibited stable NIR emission across a wide pH range (pH 3–10) and minimal nonspecific binding in vivo. In addition, Chetty et al.^94^ developed a multimodal platform by incorporating Mn^2+^ and Gd^3+^ dopants, enabling simultaneous NIR fluorescence (quantum yield ∼84%), MRI (*r*_1_ = 2.26 mM^–1^ s^–1^, *r*_2_ = 16.47 mM^–1^ s^–1^), and CT contrast (78 HU). This approach allowed real-time tracking of mesenchymal stem cells homing to melanoma models.

Despite these advantages, several limitations constrain the broader application of CuInS_2_-based QDs. The intrinsically broad emission spectra reduce spectral discrimination and limit multiplexing capacity compared to CdSe/ZnS and InP/ZnS systems. Although a cadmium-free composition improves the safety profile, long-term biocompatibility and degradation pathways remain incompletely characterized. In addition, variability in defect states can introduce heterogeneity in the optical output, complicating quantitative imaging.

For PSMA-targeted imaging in RPC, CuInS_2_/ZnS QDs offer two key translational advantages. First, the absence of cadmium removes a major regulatory barrier. Second, the large Stokes shift reduces background autofluorescence from stromal and surrounding tissues, improving contrast. Their tunable NIR emission (∼550–950 nm) overlaps with the optical transmission window of biological tissues, supporting in vivo imaging applications. However, the broad emission bandwidth (80–120 nm fwhm) limits multiplexing relative to CdSe/ZnS and InP/ZnS platforms. Furthermore, stable ligand conjugation to the ZnS shell requires careful optimization, as insufficient shell integrity can permit Cu^+^ ion leakage from the core. The degradation kinetics of CuInS_2_ QDs under physiological conditions are less well-defined than those of cadmium-based or silicon-based systems, highlighting the need for systematic pharmacokinetic and long-term safety studies before clinical translation.

### 6.4 Carbon and Graphene QDs

CQDs^25,27,95–98^ and GQDs^24,28,30^ represent a structurally distinct class of fluorescent nanomaterials that have attracted considerable attention as biocompatible alternatives to cadmium- or lead-based semiconductor QDs. CQDs are quasi-spherical, predominantly sp^3^-hybridized amorphous carbon nanoparticles (∼2–10 nm in diameter) whose photoluminescence arises from a combination of quantum confinement and surface functional groups, including carboxyl, hydroxyl, and amine moieties. In contrast, GQDs consist of one or a few graphene layers with lateral dimensions below 10 nm, with optical properties governed by π-electron conjugation, edge states, and structural order. These differences in hybridization and electronic structure determine the underlying photoluminescent mechanisms, achievable quantum yield, spectral tunability, and suitability for specific biomedical applications.

Synthesis routes for CQDs and GQDs reflect their structural differences. GQDs are typically produced via top-down fragmentation of graphite or graphene oxide through chemical oxidation, hydrothermal cutting, or electrochemical exfoliation. CQDs are generally synthesized through bottom-up approaches, such as hydrothermal carbonization of small organic precursors (e.g., citric acid, glucose, or amino acids) or pyrolysis of polymeric materials. Increasingly, renewable biomass feedstocks—including plant extracts and agricultural waste—are being explored as sustainable carbon sources, with demonstrated scalability and tunable photoluminescent properties.^99^

CQDs and GQDs exhibit several favorable characteristics, including broad excitation spectra, resistance to photobleaching relative to organic fluorophores, and good aqueous dispersibility. However, their photoluminescence quantum yields (typically 5–40%) remain substantially lower than those of optimized semiconductor QDs (>60–80%). A key limitation is restricted emission tunability into the near-infrared (NIR) region: most CQDs and GQDs emit in the blue-to-green spectral range, and efficient far-red or NIR emission requires deliberate chemical modification. Heteroatom doping (e.g., nitrogen, sulfur, or boron incorporation) can modulate the band gap structure and red-shift emission, as systematically reviewed by Kalluri et al.^75^ Nevertheless, achieving sufficiently bright emission in the NIR-II window (1000–1700 nm) for deep-tissue imaging remains a major unresolved challenge.

Several studies have demonstrated the potential of carbon-based QDs for cancer imaging and theranostics. Prasad et al.^100^ incorporated CQDs incorporated as pH-responsive gatekeepers on folate-receptor-targeted mesoporous silica nanoparticles, enabling pH-triggered drug release with concurrent fluorescence imaging. Li et al.^27^ reported CQDs engineered to mimic large neutral amino acids, enabling selective tumor uptake via LAT1 transporters following intravenous administration in mice. Nurunnabi et al.^58^ demonstrated that carboxylated, NIR-emitting GQDs distribute broadly across major organs but undergo partial renal clearance without overt acute toxicity, supporting their favorable biocompatibility profile. In addition, CQDs have been explored for photodynamic therapy sensitization, drug delivery, and fluorescence-guided intervention.^96^

For PSMA-targeted optical imaging in RPC, CQDs and GQDs offer clear translational advantages. The absence of regulated heavy metals removes a major toxicological and regulatory barrier, and the demonstrated renal clearance of small GQDs suggests a viable elimination pathway for clinically deployable probes. Furthermore, abundant surface functional groups facilitate the conjugation of PSMA-targeting ligands using standard coupling chemistries. However, two major limitations constrain their applications. First, NIR quantum yields remain insufficient for reliable deep-tissue imaging in anatomically relevant regions such as the prostate bed and pelvic lymph nodes, where imaging depths of several centimeters require both high brightness and longer emission wavelengths. Second, broad and often overlapping emission spectra limit multiplexing capability compared to the narrow, size-tunable emissions of semiconductor QDs. Accordingly, CQDs and GQDs are best positioned as low-toxicity platforms for repeated dosing applications, ex vivo imaging, and mechanistic studies rather than for high-sensitivity, multiplexed deep-tissue imaging. Continued advances in heteroatom doping, surface engineering, and structural design may enable extension into the NIR-II window; however, in their current form, carbon-based QDs do not yet match the optical performance required for multiplexed detection in RPC imaging workflows.

### 6.5 Silicon QDs

Silicon QDs (SiQDs) are nanocrystals of elemental silicon with a diamond cubic lattice structure and diameters typically in the range of 1–10 nm.^101–103^ Bulk silicon is an indirect band gap semiconductor (E_g ≈ 1.1 eV) with negligible radiative emission efficiency; however, quantum confinement at nanoscale dimensions relaxes this limitation and enables size-tunable photoluminescence spanning the visible to NIR range. For example, Romano et al.^104^ demonstrated that silicon nanocrystals with diameters of 4 nm emit at ∼735 nm, whereas 5 nm particles emit at ∼945 nm, both within the NIR-I window relevant for tissue imaging. SiQDs also exhibit long photoluminescence lifetimes (on the order of microseconds), enabling time-gated imaging strategies that suppress tissue autofluorescence and improve signal-to-background noise contrast.^104^

Surface chemistry is a critical determinant of SiQD stability and biological functionality. As-synthesized SiQDs typically possess hydrogen-terminated surfaces (Si–H bonds) that are hydrophobic and chemically reactive. Hydrosilylation—via UV-promoted or thermal addition of terminal alkenes—enables formation of covalent Si–C bonds, yielding stable monolayers with terminal functional groups (e.g., carboxyl, amine, alcohol) for ligand conjugation.^78^ Alternatively, click chemistry using nonadiyne-functionalized SiQDs allows modular attachment of azide-bearing molecules in a single step, producing particles with high resistance to photobleaching under physiological conditions.^77^ Encapsulation strategies, including phospholipid micelles or amphiphilic polymer shells, provide another route to aqueous dispersibility while preserving optical properties. For instance, Erogbogbo et al.^101^ used micelle-encapsulated SiQDs for labeling pancreatic cancer cells, while Hessel et al.^76^ demonstrated that polymer-coated silicon nanocrystals (∼2.1 nm diameter, peak emission 650 nm) maintain stable photoluminescence across a wide range of pH and ionic strengths.

A major advantage of SiQDs is their favorable biocompatibility profile. Upon surface oxidation, silicon is converted to orthosilicic acid [Si­(OH)_4_], a naturally occurring and really cleared metabolite. In vitro studies consistently report low cytotoxicity: Cheng et al.^77^ observed no detectable toxicity in HeLa cells at concentrations up to 240 μg mL^–1^, while Tu et al.^105^ reported minimal cytotoxic effects up to 1600 μg mL^–1^ for bovine serum albumin (BSA)- and PEG-coated SiQDs. Importantly, Tu et al. also demonstrated that anti-HER2-conjugated SiQDs achieved specific immunostaining of HER2-overexpressing ovarian cancer cells with negligible nonspecific binding, providing proof of concept for antibody-mediated targeting in live-cell systems. Despite these advantages, optical performance remains a limiting factor. Photoluminescence quantum yield of 45–55% can be achieved in organic solvents, but transfer to aqueous environments typically reduces yields to 5–10%,^105^ significantly constraining detection sensitivity for in vivo imaging. Additional surface passivation and core engineering strategies are therefore required to improve emission efficiency under physiological conditions.

For PSMA-targeted imaging in RPC, SiQDs represent a heavy-metal-free platform with size-tunable NIR emission, long photoluminescence lifetimes, and well-established surface conjugation chemistry. However, reduced quantum yield in aqueous environments limits their utility for detecting lesions at clinically relevant depths such as the prostate bed and pelvic lymph nodes.

### 6.6 Perovskite QDs

Metal halide perovskite QDs (e.g., CsPbBr_3_)^15,106,107^ adopt an ABX_3_ crystal structure, in which the A site is occupied by a monovalent cation (typically Cs^+^), the B site by Pb^2+^ within the PbX_6_ octahedral framework, and the X site by halide anions (Cl^–^, Br^–^, I^–^, or mixed compositions). In contrast to the predominantly covalent bonding in II–VI or III–V QDs, the Pb–X bonds are largely ionic. This bonding character gives rise to two defining—and competing—properties: near-unity photoluminescence quantum yields (PLQYs) resulting from efficient band-edge exciton recombination and pronounced susceptibility to hydrolytic degradation in aqueous environments. Emission can be tuned continuously by varying halide composition, spanning approximately 400 nm (CsPbCl_3_) to 520 nm (CsPbBr_3_) to 680–700 nm (CsPbI_3_), with additional modulation quantum confinement. Perovskite QDs exhibit the narrowest emission bandwidths among colloidal fluorophores, fwhm values as low as 22–25 nm—narrower than CdSe/ZnS (25–35 nm), InP/ZnS (40–60 nm), and CuInS_2_/ZnS (80–120 nm) systems. This exceptional spectral purity enables highly precise multiplexed imaging. Encapsulation strategies can further enhance optical performance; for example, silica/alumina-encapsulated CsPbBr_3_ QDs have achieved PLQYs approaching 90%, while CsPbBr_3_/cellulose nanocrystal hybrid films exhibit fwhm values of 22 nm and retain >99% of photoluminescence intensity under prolonged thermal and UV stress.^108,109^

Despite these favorable photophysical properties, biological application remains limited by instability in aqueous media. Zhang and Zhu^106^ noted that although perovskite QDs exhibit superior brightness and photostability under continuous illumination, rapid degradation in water remains the principal barrier to biomedical use. This instability arises from the dissociation of halide ions from the Pb–X lattice upon exposure to polar solvents, leading to structural collapse and fluorescence quenching within minutes to hours in the absence of robust encapsulation.^110^ Encapsulation and composite strategies have been developed to mitigate these effects. Wang et al.^111^ encapsulated CsPbBr_3_ QDs within poly­(methyl methacrylate) (PMMA) nanospheres (∼112 nm), achieving a PLQY of 73% and retaining 81% of fluorescence after 80 days in aqueous media, with no detectable cytotoxicity in HeLa cells. Zhang et al.^106^ demonstrated targeted fluorescence imaging using CsPbBr_3_ QDs functionalized with azithromycin (AZI) and a CD44v6-targeting peptide, achieving selective labeling of gastric cancer cells and tumor accumulation in xenograft models without observable short-term toxicity. In addition, Ryu et al.^107^ exploited the high X-ray attenuation coefficient of Pb to develop dual-modality imaging probes based on CsPbBr_3_ QDs encapsulated in SiO_2_ and conjugated with anti-CD44 antibodies, enabling real-time X-ray imaging of pancreatic tumors at low doses.

However, these strategies introduce new trade-offs. Encapsulation substantially increases the hydrodynamic diameter, which may limit renal clearance and alter biodistribution. Although double-layer SiO_2_ encapsulation reduces Pb leakage to very low levels (e.g., 0.2 ppb over 14 days), stability can be compromised under certain conditions such as alkaline pH. Liu et al. achieved water dispersibility by coating CsPbBr_3_–Fe_3_O_4_ nanocomposites with stearic acid and amphiphilic DSPE-mPEG, retaining 42.8% of the initial fluorescence after 37 days in an aqueous medium while adding T_2_ MRI contrast capability.^112^ A second major barrier is the presence of Pb^2+^ in high-performance perovskite QDs. Lead toxicity presents a regulatory challenge that is at least as significant as, and in some respects more restrictive than, that associated with cadmium-based systems, given the well-established neurotoxicity and nephrotoxicity of inorganic lead. Although lead-free alternatives, including tin (CsSnX_3_), bismuth (Cs_3_BiX_6_, Cs_2_AgBiX_6_), and double-perovskite systems, are under active development, these currently exhibit lower PLQYs and broader emission spectra, reducing their competitiveness with CsPbX_3_ systems.^15^

For PSMA-targeted imaging in RPC, perovskite QDs present a clear paradox. They offer the highest optical precision among QD platforms, with exceptional brightness and the narrowest emission bandwidths for spectral multiplexing, yet remain the least physiologically mature. Their primary emission wavelengths (400–520 nm for CsPbCl_3_ and CsPbBr_3_) lie outside the optimal NIR window (700–900 nm) for deep-tissue imaging, while CsPbI_3_, capable of emission near 700 nm, exhibits poor phase stability at room temperature. Encapsulation strategies required for aqueous stability increase particle size, potentially limiting lymphatic transport and renal clearance. Notably, no PSMA-targeted perovskite QD systems have been reported to date, and the regulatory pathway for lead-containing nanomaterials in oncology remains highly uncertain.

### 6.7 Positioning Material Choice within Imaging Workflows

Surface chemistry plays a central yet material-dependent role in determining both the optical performance and biological behavior of QDs. In metallic semiconductor QDs, including Cd-, In-, and Cu-based systems, surface passivation through core–shell architectures (e.g., ZnS) and ligand engineering is essential to stabilize the nanocrystal, suppress surface trap states, and limit ion leakage that can contribute to cytotoxicity. In contrast, nonmetallic QDs—such as carbon-, graphene-, and silicon-based platforms—are less dependent on passivation against core degradation. Instead, their optical properties and biocompatibility are governed primarily by intrinsic composition and surface functional groups. Consequently, surface engineering in metallic QDs is directed toward preserving photophysical performance while mitigating toxicity, whereas in nonmetallic systems, it predominantly regulates solubility, targeting capability, and interactions with biological environments.

Across the material platforms reviewed, no single QD system simultaneously maximizes the optical performance, biocompatibility, and translational feasibility. Cadmium-based QDs define the benchmark for brightness, spectral tunability, and multiplexing capability and, therefore, remain indispensable for preclinical imaging and mechanistic studies. Cadmium-free semiconductor QDs, including InP- and CuInS_2_-based systems, provide a compromise between optical performance and improved safety profiles, supporting their use in translational and in vivo imaging contexts. In contrast, carbon-, graphene-, and silicon-based QDs offer superior biocompatibility and more favorable clearance characteristics but exhibit a reduced quantum yield and limited multiplexing capability.

Accordingly, material selection should be guided by the specific imaging context rather than by optical metrics alone. High-brightness semiconductor QDs are best suited for applications requiring maximal sensitivity and multiplexing such as detailed mechanistic studies or high-content tissue imaging. In contrast, carbon- and silicon-based QDs are more appropriate for applications prioritizing safety, including longitudinal or repeated dose studies. Intermediate platforms, such as InP- and CuInS_2_-based QDs, may offer the most practical balance for preclinical in vivo imaging, where both performance and tolerability are required.

This application-driven framework aligns material properties with the spatial scale, depth, and biological constraints of imaging workflows, as outlined in Figure 5 and Tables 1 and 4. Such positioning is essential for translating QD technologies from experimental probes to clinically relevant tools and for bridging the gap between whole-body imaging and high-resolution molecular analysis in RPCs.

## 7 Toxicity, Biodistribution, and Translational Barriers

The translational potential of QDs in PC imaging is determined less by their optical performance than by material-dependent toxicity, in vivo fate, and feasibility of regulatory approval.^81,82,113–115^ Across QD platforms, biological risk arises from an interplay between core composition, surface chemistry, colloidal stability, and residence time within clearance organs. These factors collectively govern biodistribution, degradation, and long-term tissue interactions. Accordingly, evaluation of translational readiness must extend beyond short-term cytotoxicity assays to encompass pharmacokinetics, organ accumulation, persistence, and clearance pathways.

### 7.1 Composition-Dependent Toxicity

Core composition is a major determinant of QD-associated toxicity, although it does not act in isolation from surface chemistry and colloidal stability. Cadmium-based QDs, despite their superior optical performance, present a well-recognized risk due to potential Cd^2+^ ion release following core degradation or intracellular processing. Such release can induce oxidative stress, mitochondrial dysfunction, and reduced cellular viability, particularly under prolonged exposure or repeated dosing. Core–shell architectures and surface passivation substantially mitigate these effects; however, cadmium-containing QDs remain subject to heightened scrutiny for in vivo and clinical applications.

Cadmium-free semiconductor QDs, including InP- and Cu InS_2_-based systems, have been developed as lower-toxicity alternatives. These materials generally exhibit reduced acute toxicity compared with cadmium-based QDs, but their safety profiles remain strongly dependent on surface chemistry, structural stability, and degradation behavior.^89–91^ Importantly, the absence of cadmium does not imply biological inertness. Potential release of indium- or copper-containing species, as well as long-term accumulation in clearance organs, necessitates careful evaluation on a case-by-case basis.^83^

Carbon-based QDs, including CQDs and GQDs, exhibit comparatively favorable biocompatibility profiles due to their nonmetallic composition and reduced risk of ion release.^29,95–97^ These properties make them attractive for repeated or longitudinal imaging applications. However, their lower optical performance limits utility in high-sensitivity or deep-tissue imaging contexts. In contrast, perovskite QDs, while offering exceptional brightness and spectral precision, face significant translational barriers due to intrinsic chemical instability and presence of toxic elements (e.g., Pb^2+^) in many formulations.^15,106,113^ Although encapsulation strategies can mitigate these risks, they remain under active development and have not yet fully addressed concerns regarding long-term safety.

### 7.2 Biodistribution and Persistence

Independent of core composition, systemically administered nanoparticles, including QDs, exhibit characteristic biodistribution patterns governed by hydrodynamic diameter, surface charge, and the dynamic protein corona that forms rapidly upon entry into the bloodstream.^116,117^ Uptake by the MPS typically leads to rapid accumulation in the liver and spleen, often within minutes to hours after administration, with retention persisting for days to weeks, depending on particle size and surface chemistry.^64,117^ Additional distribution to the kidneys, lymphatic tissues, or bone marrow may occur, particularly for smaller or surface-modified QDs. Quantitative pharmacokinetic studies illustrate the magnitude and surface dependence of these effects. PEG-coated NIR-II-emitting Ag_2_Se QDs exhibit a blood half-life of approximately 0.4 h following intravenous administration in mice, with progressive biotransformation into elemental silver and selenium species and gradual elimination via fecal and urinary routes within approximately 1 week, without overt acute systemic toxicity.^56^ In contrast, uncoated semiconductor QDs demonstrate rapid clearance from circulation and pronounced organ accumulation: bare CdSe/ZnS nanocrystals are removed from the bloodstream within minutes and accumulate in the liver at levels exceeding 50% of the injected dose per gram of tissue, creating a persistent intracellular reservoir from which Cd^2+^ ions may be released upon core degradation.^49,118^ Surface PEGylation extends circulation time from minutes to hours in a chain-length- and density-dependent manner but does not eliminate MPS uptake, with accumulation remaining predominantly hepatosplenic.^49^

Hydrodynamic diameter relative to the renal filtration threshold is a critical determinant of clearance. QD with hydrodynamic diameters below approximately 5.5 nm and neutral or zwitterionic surface coatings can undergo efficient glomerular filtration and urinary excretion, providing a “clearance-by-design” pathway that substantially reduces the long-term organ retention.^57^ This size-and-surface relationship represents a key engineering target for translational probe development. In tumor-bearing models, the enhanced permeability and retention (EPR) effect can increase passive tumor accumulation, as reported for graphene QDs and semiconductor platforms;^58^ however, hepatosplenic accumulation typically remains quantitatively dominant and should not be interpreted as evidence of selective tumor targeting. Representative pharmacokinetic trends across QD platforms and surface chemistries are summarized in Table 4.

These biodistribution patterns have direct implications for the translational development of PSMA-targeted QD probes. Claims of biocompatibility must be supported by time-resolved pharmacokinetic studies, quantitative organ burden measurements at multiple postinjection time points, and histopathological analyses, rather than short-term cytotoxicity assays alone. Acute in vitro assays are insufficient to capture deposition kinetics, biotransformation pathways, and chronic organ exposure—parameters that are central to regulatory risk assessment, particularly for systemically administered or repeat-dose imaging agents. Notably, as of 2026, no PSMA-targeted QD probe has advanced to phase I clinical evaluation. This gap reflects not a lack of optical proof of concept but the substantial translational barrier between preclinical imaging performance and the safety, pharmacokinetic characterization, and manufacturing consistency required for Investigational New Drug (IND) approval. Moreover, incomplete clearance remains a persistent challenge, with a fraction of administered QDs retained in clearance organs for extended periods (weeks to months), particularly for particles exceeding renal filtration thresholds. These findings underscore the importance of designing QDs with controlled size, surface properties, and degradability to promote efficient clearance and minimize long-term accumulation.

### 7.3 Mitigation Strategies: Surface Engineering and Clearance-by-Design

Across QD platforms, mitigation of biological risk is most effectively achieved through stabilization of the nanocrystal core and control of biological identity via surface engineering. Core–shell architectures (e.g., ZnS passivation) reduce ion leakage and suppress surface trap states, thereby preserving optical performance while limiting toxicity. Complementary strategies—including polymer coatings, PEGylation, and tailored ligand chemistries—enhance colloidal stability, reduce nonspecific protein adsorption, and modulate cellular uptake by influencing protein corona formation.

Increasing emphasis has been placed on “clearance-by-design” approaches, which aim to minimize long-term organ retention by controlling hydrodynamic size, surface charge, and aggregation behavior.^26,27^ QDs engineered with small effective diameters and stable, hydrophilic, and near-neutral (e.g., zwitterionic) surface coatings are more likely to undergo renal or hepatobiliary clearance, thereby reducing persistence in clearance organs. These considerations underscore that surface engineering is not solely a means of improving stability or targeting but a central determinant of in vivo fate. For optical probes intended for repeated or longitudinal imaging, rational integration of size control, surface passivation, and ligand design is essential to balance imaging performance with safe clearance profiles.

### 7.4 Regulatory Perspective and Practical Positioning

From a regulatory perspective, QD-based probes face substantially higher barriers to approval than conventional small-molecule fluorophores, particularly when heavy metals are present or systemic administration is required.^119^ Given these constraints, the most realistic near-term translational applications for QDs are those in which systemic exposure can be minimized and clinical benefits are clearly defined. These include ex vivo tissue analysis, high-resolution validation of molecular targets, intraoperative imaging for margin assessment, and preclinical imaging to support the therapeutic development.

Cadmium-free semiconductor QDs and carbon-based platforms offer potential pathways toward safer optical probes for repeated or longitudinal use; however, even these systems require rigorous and standardized safety characterization including long-term biodistribution and clearance studies. Ultimately, successful translation depends on aligning material selection, surface engineering, and imaging application with realistic regulatory expectations rather than maximizing optical performance in isolation.

## 8 Overall Perspective: QDs at the Translational Frontier

Among the material platforms reviewed, InP/ZnS and CuInS_2_/ZnS QDs currently represent the most mature cadmium-free semiconductor systems. InP/ZnS QDs routinely achieve photoluminescence quantum yields of 30–70%, with emission tunable into the NIR-I window and, in some cases, extending toward the NIR-II region. CuInS_2_/ZnS QDs, by contrast, exhibit broad emission arising from donor–acceptor recombination, which can be advantageous for multimodal detection, although it complicates spectral unmixing in multiplexed imaging. Neither platform exhibits the acute Cd^2+^ ion release that remains the principal toxicological limitation of CdSe-based QDs, and both have demonstrated PSMA-targeted functionality in preclinical models. Carbon and silicon QDs offer clear advantages in biocompatibility; however, they do not yet achieve the NIR photoluminescence efficiencies required for deep-tissue, multiplexed imaging of RPC. Perovskite QDs occupy the opposite extreme, providing exceptional optical performance but remaining constrained by intrinsic instability in aqueous environments and the unresolved toxicological challenges associated with lead-containing compositions.

Three major technical bottlenecks define the gap between current capabilities and a clinically translatable PSMA-targeted QD probe. First, efficient NIR-II emission (1000–1700 nm) has not yet been achieved in heavy-metal-free QD systems without substantial loss of quantum yield. Second, maintaining a total hydrodynamic diameter below the renal filtration threshold (∼5.5 nm)^57^ while incorporating shell passivation, surface coatings, and targeting ligands remains an unsolved engineering challenge. Third, the absence of validated, good manufacturing practice (GMP)-compliant synthesis and functionalization protocols limits regulatory progression as significantly as optical or pharmacokinetic constraints.

When compared to alternative optical probes, QDs retain distinct photophysical advantages. Relative to small-molecule NIR fluorophores such as indocyanine green (ICG) or IRDye 800CW, QDs offer superior photostability, higher per-particle brightness, and narrower emission bandwidths that support multiplexed imaging. Compared with aggregation-induced emission (AIE) luminogens, QDs similarly provide greater spectral precision and tunability, although recent advances in NIR-II AIE probes have reduced this gap.^120,121^ However, these advantages must be considered alongside persistent limitations, including larger hydrodynamic size, MPS sequestration, and the absence of established regulatory pathways. In contrast, small-molecule fluorophores remain clinically dominant because of their predictable pharmacokinetics and well-defined approval frameworks.

Accordingly, the most realistic near-term translational applications of PSMA-targeted QDs are not whole-body systemic imaging for initial staging or localization of biochemical recurrence, where PSMA PET/CT already performs at a level that optical probes cannot plausibly match at tissue depths beyond a few millimeters (Table 2).^23,122^ Rather, the highest-value applications are likely to be localized and high resolution. These include ex vivo fluorescence analysis of surgical resection margins immediately following radical prostatectomy, where QDs can provide durable signal readout and enable multimarker tissue assessment. Positive surgical margins occur in approximately 15–22% of robot-assisted radical prostatectomy cases and are associated with approximately 2-fold increased risk of biochemical recurrence,^123,124^ highlighting the clinical relevance of such approaches in the absence of systemic exposure. Additional applications include intraoperative fluorescence guidance during robot-assisted radical prostatectomy for the identification of PSMA-expressing lymph nodes within the surgical field, where limited tissue depth and localized probe administration reduced regulatory burden. QDs are also well suited for high-resolution preclinical validation of PSMA expression heterogeneity in patient-derived xenograft models, where they can provide mechanistic insights that inform the design of clinically translatable imaging probes.

## 9 Future Directions in Targeted Cancer Therapy Workflows

The future impact of QDs in oncology will be defined by their ability to provide actionable molecular information at spatial scales that complement established clinical imaging modalities while meeting practical requirements for safety, clearance, and regulatory feasibility. In this context, QDs are best positioned not as standalone clinical diagnostics but as enabling probes that strengthen targeted cancer therapy workflows through localized biomarker mapping and mechanistic insights.

Several near- and mid-term application domains warrant particular attention for RPC. First, in preclinical and translational research, QD-enabled multiplexed imaging can dissect tumor heterogeneity, adaptive resistance, and biomarker dynamics over time. By enabling simultaneous visualization of PSMA alongside complementary molecular markers—including androgen receptor splice variants and resistance-associated phenotypes—QDs support a more nuanced understanding of disease evolution and therapeutic response than single-target imaging approaches.

As outlined in the preceding section, ex vivo margin assessment and intraoperative lymph node identification represent the most immediately actionable translational contexts for PSMA-targeted QD probes, as the tissue depth is limited and systemic exposure is minimized. Beyond these procedural settings, integration with patient-derived models—including organoids, explant cultures, and organ-on-chip platforms—offers a scalable and biologically tractable pathway for deploying QDs in precision oncology research. Within these systems, QDs can serve as quantitative reporters of molecular binding, cellular uptake, and microenvironment-dependent phenotypes, enabling controlled investigation of PSMA expression dynamics, adaptive resistance mechanisms, and phenotypic heterogeneity at subcellular resolution. Such applications bridge fundamental cancer biology and translational probe development without requiring the regulatory infrastructure associated with systemic deployment.

A further application domain is theranostic integration, in which QDs function simultaneously as optical reporters and phototherapeutic agents. NIR-II-emitting probes with combined fluorescence imaging and photothermal or photodynamic therapeutic capabilities have been demonstrated with AIE-based systems,^125^ Analogous strategies are feasible for QDs and are particularly well suited to PSMA-targeted delivery, where ligand-mediated specificity can concentrate phototherapeutic energy at tumor sites. In intraoperative or endoscopic settings—where illumination can be precisely controlled and tissue access is direct—PSMA-targeted QD theranostic probes could enable simultaneous visualization and localized treatment of residual disease within a single procedure. This paradigm is distinct from purely diagnostic optical imaging and represents a high-value application not currently addressed by existing clinical platforms.

Ultimately, the primary constraint on the translation of QDs into targeted cancer therapy workflows is not optical performance or molecular targeting specificity—both of which continue to improve—but the generation of regulatory-grade evidence. Progress will require systematic characterization of long-term biodistribution, organ burden, and clearance kinetics across QD material classes; development of IND-enabling toxicology data sets in relevant animal models; and establishment of GMP-compliant synthesis and functionalization protocols that ensure reproducible manufacturing at clinical scale. Successful translation will depend on aligning QD probe design with clearly defined clinical needs at the interface of molecular specificity and cellular-scale resolution and on adopting staged deployment strategies—from rigorous preclinical validation to ex vivo implementation and carefully controlled first-in-human studies—that generate the safety and performance data required for regulatory approval.

## 10 Conclusion

Targeted cancer therapy increasingly depends on precise molecular detection to guide the diagnosis, patient stratification, treatment selection, and therapeutic monitoring. In RPC, PSMA-targeted PET imaging has transformed clinical practice by enabling the sensitive whole-body detection of recurrent disease. However, its intrinsic spatial resolution and limited multiplexing capacity restrict the ability to interrogate cellular-scale heterogeneity and micrometastatic processes that drive disease progression and the treatment response.

QDs provide a complementary optical platform capable of addressing this limitation across preclinical and translational settings. Their distinctive photophysical properties enable sensitive detection of low-abundance targets and high-resolution analysis of heterogeneous tumor cell populations, particularly in contexts in which conventional imaging modalities lack sufficient spatial or molecular resolution. While cadmium-based QDs remain the benchmark for optical performance, the growing development of cadmium-free semiconductor- and carbon-based platforms offers promising pathways toward improved translational feasibility.

Importantly, QDs are unlikely to replace established clinical imaging modalities in oncology. Instead, their greatest value lies in augmenting targeted cancer therapy workflows by bridging the resolution gap between whole-body imaging and cellular-level biology. Continued progress in materials design, surface engineering, and safety characterization—together with the identification of clinically relevant use cases—will be essential for advancing QD-based optical probes from high-performance research tools to translational technologies that contribute meaningfully to precision cancer management.

## Abbreviations

Cd^2+^: cadmium ion
CdSe: cadmium selenide
CdSe/ZnS: cadmium selenide/zinc sulfide core–shell quantum dots
CT: computed tomography
InP: indium phosphide
MRI: magnetic resonance imaging
NIR: near-infrared
NIR-II: near-infrared II
PC: prostate cancer
PEG: polyethylene glycol
PET: positron emission tomography
PLQY: photoluminescence quantum yield
PQDs: perovskite quantum dots
PSA: prostate-specific antigen
PSMA: prostate-specific membrane antigen
QD: quantum dot
QY: photoluminescence quantum yield
fwhm: full-width at half-maximum
DAP: donor–acceptor pair
AIE: aggregation-induced emission
MPS: mononuclear phagocyte system
HD: hydrodynamic diameter
EPR: enhanced permeability and retention
GMP: good manufacturing practice
IND: Investigational New Drug
CQD: carbon quantum dot
GQD: graphene quantum dot
SiQD: silicon quantum dot
RPC: recurrent prostate cancer
ZnS: zinc sulfide
